# Iron Regulates Cellular Proliferation by Enhancing the Expression of Glucose Transporter GLUT3 in the Liver

**DOI:** 10.3390/cells13131147

**Published:** 2024-07-04

**Authors:** Kleber S. Ribeiro, Eshani Karmakar, Christine Park, Richa Garg, George P. Kung, Isha Kadakia, Jyotsna S. Gopianand, Tejas Arun, Oleg Kisselev, Jaya P. Gnana-Prakasam

**Affiliations:** School of Medicine, Saint Louis University, St. Louis, MO 63104, USAeshani.karmakar@health.slu.edu (E.K.); richa.garg@health.slu.edu (R.G.);

**Keywords:** iron overload, GLUT3, AMPK, CREB1, cell proliferation

## Abstract

Iron is often accumulated in the liver during pathological conditions such as cirrhosis and cancer. Elevated expression of glucose transporters GLUT1 and GLUT3 is associated with reduced overall survival in patients with hepatocellular carcinoma. However, it is not known whether iron can regulate glucose transporters and contribute to tumor proliferation. In the present study, we found that treatment of human liver cell line HepG2 with ferric ammonium citrate (FAC) resulted in a significant upregulation of GLUT3 mRNA and protein in a dose-dependent manner. Similarly, iron accumulation in mice fed with high dietary iron as well as in mice injected intraperitoneally with iron dextran enhanced the GLUT3 expression drastically in the liver. We demonstrated that iron-induced hepatic GLUT3 upregulation is mediated by the LKB1/AMPK/CREB1 pathway, and this activation was reversed when treated with iron chelator deferiprone. In addition, inhibition of GLUT3 using siRNA prevented iron-mediated increase in the expression of cell cycle markers and cellular hyperproliferation. Furthermore, exogenous sodium beta-hydroxybutyrate treatment prevented iron-mediated hepatic GLUT3 activation both in vitro and in vivo. Together, these results underscore the importance of iron, AMPK, CREB1 and GLUT3 pathways in cell proliferation and highlight the therapeutic potential of sodium beta-hydroxybutyrate in hepatocellular carcinoma with high GLUT3 expression.

## 1. Introduction

Iron serves as an essential micronutrient crucial for numerous cellular functions such as oxygen transport, energy metabolism, and DNA synthesis [1]. However, imbalances in iron regulation can lead to pathological conditions during genetic iron overload disorders such as hereditary hemochromatosis and secondary iron overload associated with repeated blood transfusions or excessive dietary intake [2]. Iron overload primarily affects the liver as it is the primary site of iron storage in the body [3]. Given the liver’s pivotal role in the body’s glucose homeostasis [4], hepatic iron overload contributes to metabolic dysregulation, fostering conditions such as insulin resistance, diabetes mellitus, and non-alcoholic fatty liver disease, largely attributed to oxidative stress or impaired autophagy [5,6,7]. Similarly, mounting evidence suggests that iron overload potentiates cellular proliferation and hepatocellular carcinoma (HCC) [8]. However, the mechanisms by which iron contributes to tumor progression through metabolic reprogramming is not clearly understood.

Glucose, the primary metabolic energy source obtained from the diet, is absorbed by enterocytes through sodium coupled glucose transporter 1 (SGLT1) [9] and released into the blood stream through glucose transporter 2 (GLUT2) [10]. The expression patterns of the critical isoforms of class 1 facilitative glucose transporters (GLUTs) differ in tissue distribution based on the metabolic demands and glucose requirements of a specific cell type. GLUT1, for instance, is expressed in most of the cell types at a basal level, with the highest expression found in erythrocytes [11]. Conversely, GLUT2 is highly expressed in the liver, pancreatic β cells, and enterocytes [12], while GLUT3 predominates in the neuronal cells [13], and GLUT4 is primarily expressed in insulin-sensitive tissues such as skeletal muscles, cardiomyocytes, and adipocytes [14]. Since iron is stored primarily in the liver, it is critical to determine whether iron regulates the expression of glucose transporters in the liver.

Aerobic glycolysis, also known as the Warburg effect, is one of the hallmarks of cancer cells characterized by increased dependence on glucose to generate energy primarily through glycolysis even in the presence of oxygen, in contrast to normal cells that produce energy predominantly through oxidative phosphorylation [15]. Consequently, numerous studies underscore the increased vulnerability of cancer cells to apoptosis under conditions of glucose deprivation compared to normal cells, with inhibition of glucose transport resulting in diminished tumor proliferation [16,17,18]. Interestingly, enhanced expression levels of glucose transporters GLUT1 and GLUT3 have been consistently observed across various cancers [18,19], including hepatocellular carcinoma [20,21]. Since iron accumulation is associated with hepatocellular carcinoma, this study aimed to elucidate whether iron regulates the expression of glucose transporters in the liver and their involvement in cellular hyperproliferation.

## 2. Materials and Methods

### 2.1. Animals

C57BL6/J mice were obtained from Jackson Laboratory (Bar Harbor, ME, USA) and maintained at the animal facility of Saint Louis University School of Medicine. Age-, gender-, and weight-matched mice were randomly divided into different treatment groups with 4–6 mice per experimental group. Systemic iron overload was induced in mice either by intraperitoneal injection of iron dextran or using a 2% carbonyl iron diet. For the iron dextran (Fe-Dex) experiment, 6- to 8-week-old C57BL/6J mice were injected intraperitoneally once every week with iron dextran (1 g/kg body weight) for 6 weeks to induce iron overload similar to the procedure described previously [22]. Phosphate-buffered saline (PBS) was injected in the control mice. For the dietary iron overload experiment, mice were fed with either a standard iron diet (SD, 48 ppm iron, TD.80394; Harlan Laboratories, Indianapolis, IN, USA) or a high-iron diet (HID, 2% carbonyl iron, TD.08496; Harlan Laboratories) from 4 weeks of age until they were sacrificed at 16 weeks of age. For β-hydroxybutyrate (BHB) treatment, mice were administered with 250 mg/kg of sodium BHB (Sigma Aldrich, St. Louis, MO, USA, Cat # 54965) intraperitoneally on alternate days from 8 weeks until they were sacrificed at 12 weeks [23]. All procedures involving mice with protocol number 2844 were approved by the Saint Louis University Institutional Committee on Animal Use for Research and Education.

### 2.2. Cell Culture and Treatments

HepG2 cells were grown in Dulbecco Modified Eagle Medium (DMEM, Gibco, Grand Island, NY, USA, Cat #11995-065) supplemented with 10% FBS and 100 U/L Penicillin/Streptomycin (Gibco, Cat #15140-122) and maintained in a humidified chamber with 5% CO_2_ at 37 °C. Cells were seeded and used for biochemical experiments after reaching 90% confluency followed by a 6 h serum starvation before treatments were carried out. Ferric ammonium citrate (FAC) (250 μg/mL; Sigma Aldrich, Cat #F5879) [24,25] was used to simulate iron overload in vitro in cell culture followed by treatment with or without iron chelator deferiprone (DFP) (100 μM; Sigma Aldrich, Cat #379409) [26] or sodium BHB (800 μM; Sigma Aldrich, Cat #54965) for 24 h within the dosage and time range used previously in the literature [27,28].

### 2.3. siRNA Transfection

Cells seeded uniformly in a 6-well plate were left overnight to grow and attain nearly 60% confluency for performing GLUT3 siRNA transfection next day. Lipofectamine-2000 (Invitrogen, Waltham, MA, USA, Cat #11668-019) reagent was used for transfection according to the manufacturer’s protocol. Transfection was performed using a concentration of 20 nM of GLUT3-specific siRNA (Santa Cruz Biotechnology, Dallas, TX, USA, Cat #sc-41218) for 5 h in the HepG2 cells. Then, 48 h post transfection in normal medium, cells were treated with FAC for 24 h and harvested for the downstream experiments as described previously [29]. The control cells were transfected with scrambled siRNA (Santa Cruz Biotechnology, Cat #sc37007). Inhibition of GLUT3 expression was confirmed by real-time PCR and Western blot.

### 2.4. RNA Isolation, cDNA Synthesis, and Real-Time PCR

Total RNA was extracted from the cells using Trizol reagent (Invitrogen, Cat# 15596026). The extracted RNA was quantified by measuring the absorbance at 260 nm. In addition, 500 ng RNA was used for cDNA preparation using Superscript IV VILO^TM^ Master Mix (Invitrogen, Cat# 11766050) following the manufacturer’s protocol. Relative expression of specific genes was quantified by the ABI Quant Studio3 real-time PCR system using PowerUp^TM^ SYBR^TM^ Green Master Mix (Applied Biosystems, Waltham, MA, USA, Cat# A25742) and normalized with the housekeeping 18S rRNA expression. Data were plotted as fold change of a given target gene in treated samples compared with their corresponding control cells or tissue. The list and sequence of the specific human and mouse primers used are provided in Supplementary Table S1.

### 2.5. Protein Extraction and Immunoblotting

Protein extraction from the cells or tissues was performed using RIPA Lysis buffer (ThermoScientific, Waltham, MA, USA, Cat# 89900) supplemented with DTT, PMSF, and protease/phosphatase inhibitor cocktail 100× (Cell Signaling, Danvers, MA, USA, Cat #5872S). The concentration of protein was estimated by a bicinchoninic acid assay (Thermo Fisher Scientific, Waltham, MA, USA, Cat #23227); 30 μg of protein was subjected to SDS-polyacrylamide gel electrophoresis and transferred onto the nitrocellulose membrane. Then, the membrane was blocked for 1–2 h with 5% bovine serum albumin (fatty acid free) or 5% non-fat milk and incubated with the primary antibodies overnight at 4 °C. The next day, blots were washed with 1XTBST at least 3 times and incubated with a secondary antibody for about 90 min at room temperature (RT). The blots were washed with TBST another 3 times. The blots were incubated with ECL substrate (Bio-Rad, Hercules, CA, USA, Cat #170-5061) and the bands were detected using a chemiluminescence detection system (iBright FL1500 Imaging System, Thermo Fisher). The following primary antibodies were used: Phospho-AMPK (Cell signaling, Cat #2531S, dilution: 1:1000), Phospho-CREB (Invitrogen, Cat #MA5-11192, dilution 1:1000), GLUT3 (Invitrogen, Cat #MA5-32087, dilution 1:2000), cyclin D1 (Cell Signaling, Cat #2978S), α-tubulin (Abcam, Cambridge, UK, Cat #4074), and β-actin (Abcam, Cat #6276) at 1:1000 dilutions. Corresponding anti-rabbit (Cell Signaling, Cat #7074S) and anti-mouse (Cell Signaling, Cat #7076S) HRP-linked secondary antibodies were used.

### 2.6. Immunoprecipitation

Immunoprecipitation (IP) was performed using a Pierce Magnetic IP/Co-IP kit (ThermoScientific, Cat #88804) following a standard protocol [30]. HepG2 cells were lysed using RIPA buffer supplemented with a protease/phosphatase inhibitor cocktail. Subsequently, cell lysates were incubated with the primary antibody LKB1 (Cell Signaling, Cat #3047S) overnight at 4 °C followed by incubation with Protein A/G magnetic beads. The immunocomplexes were washed following the IP kit protocol and subjected to Western blotting with an anti-acetylated lysine antibody (Cell Signaling, Cat #9441S).

### 2.7. Immunostaining

Immunohistochemistry in frozen liver tissue sections was performed as described previously [31]: 4% paraformaldehyde-fixed tissue slides were incubated for 90 min in a blocking solution of 5% bovine serum albumin (BSA; Sigma Aldrich, St. Louis, MO, USA, Cat A7906) followed by incubation with primary antibody GLUT3 (Invitrogen, Cat #MA5-32087, dilution 1:400) or TFR1 (Abcam, Cat #84036, dilution 1:400) overnight at 4 °C in a humified chamber. Negative control sections involved omission of the primary antibodies. The next day, the slides were washed and incubated for 90 min with fluorescence-tagged Alexa Fluor ^TM^ 488 (Invitrogen, Cat #A11008) or Alexa Fluor^TM^ 647 (Invitrogen, Cat #A31573) secondary antibodies. For nucleus staining, slides were incubated with Hoechst solution for 5–7 min and washed. Following this, slides were mounted with coverslips using the Prolong^TM^ Diamond Antifade Mountant (Invitrogen, Cat #P36970). Visualization and acquisition of fluorescent images were carried out using a Leica SP8 confocal microscope (Leica Microscope Systems, Wetzlar, Germany).

For the immunostaining of cells, 6-well plates were used to seed cells on a glass coverslip and maintained overnight at 37 °C in a humidified chamber with 5% CO_2_. The next day, the cells were kept in serum-free DMEM media for 6–8 h. The cells were then pretreated with DFP or BHB followed by FAC treatment for 24 h. Post treatment, cells were washed with PBS and fixed using 4% paraformaldehyde for 10 min and again washed with PBS 3 times. This step was followed by permeabilization with 0.1% TritonX-100 for 5 min. Cells were then incubated in a blocking solution of 3% BSA for 90 min. Subsequently, cells were incubated with the primary antibody overnight in a humified chamber and then washed with PBS. Then, cells were incubated with a fluorescence-tagged secondary antibody for 90 min and then washed thrice with PBS. Cells were then stained with 0.25 μg/mL DAPI nuclear stain for 5 min. Coverslips were then mounted with Prolong^TM^ Diamond Antifade Mountant and cells were examined using a Leica SP8 confocal microscope.

### 2.8. Cell Proliferation Assays

The effect of iron in the presence or absence of GLUT3 siRNA treatment on HepG2 cell proliferation was quantified using a colorimetric MTT (3-(4,5-dimethyl-2-thiazolyl)-2,5-diphenyl-2H-tetrazolium bromide) assay as described previously [32] or by a colorimetric BrdU (bromodeoxyuridine) assay. Cells were seeded in a 96-well plate at a density of 10,000 cells/well. Post-siRNA transfection and 72 h FAC treatment, cells were incubated for 4 h at 37 °C with 5 mg/mL MTT (Millipore Sigma, Burlington, MA, USA, Cat #M5655) or with BrdU (BrdU Cell Proliferation ELISA kit, Abcam, Cat #ab126556) for 24 h. For the MTT assay, the formazan crystals formed post-MTT incubation in the viable cells were solubilized with DMSO and absorbance was read at 570 nm. Data are represented as percentage cell viability. For the BrdU assay, incorporation of BrdU in the newly synthesized DNA of actively proliferating cells was quantified according to the kit manufacturer’s protocol.

### 2.9. Statistical Analysis

All statistical analysis was performed using GraphPad Prism 10 software. Data are expressed as mean ± SEM. Statistical significance between two groups was determined using a two-tailed Student’s *t*-test or one-way ANOVA (followed by Bonferroni analysis) when the number of groups compared was more than two. All the experiments were performed in triplicates. The values of *p* < 0.05 were considered significant.

## 3. Results

### 3.1. Iron Upregulates Hepatic GLUT3 Expression

**Figure 1:**
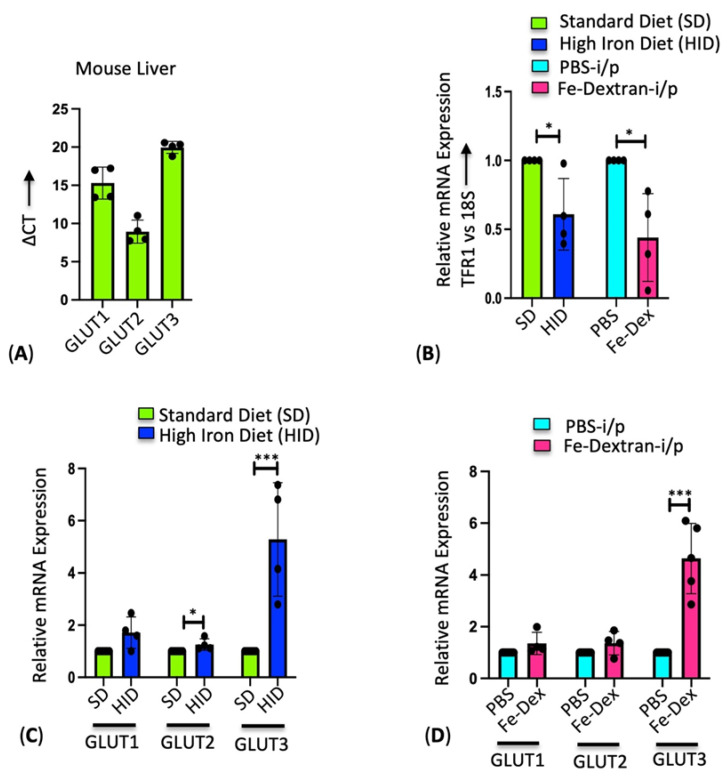
Differential expression of GLUTs upon induction of iron overload in the liver. (**A**) Comparison of the ΔCT values for GLUT1, GLUT2, and GLUT3 expression in the mouse liver. (**B**) Relative mRNA expression of TFR1 by RT-PCR in the liver tissue of SD- or HID-fed mice and PBS- or Fe-Dex-injected mice. (**C**) Relative mRNA expression of GLUT1, GLUT2, and GLUT3 by RT-PCR in the liver tissues of SD- and HID-fed mice. (**D**) Relative mRNA expression of GLUT1, GLUT2, and GLUT3 by RT-PCR in the liver tissues of PBS- and Fe-Dex-injected mice; 18S RNA was used as an internal control. Data expressed as mean ± SEM; *n* = 4–5 mice per group; *** *p* < 0.0001, * *p* < 0.05, ns—not significant.

We first determined the expression of critical glucose transporters in the mouse liver and found GLUT2 to be expressed at the highest levels similar to the literature evidence [14], followed by GLUT1 and GLUT3, as indicated by the lowest delta Ct value for GLUT2 compared to GLUT1 and GLUT3 (Figure 1A). We checked the effect of iron on the expression of glucose transporters using mice on dietary iron overload or mice injected with iron dextran. We first confirmed the iron overload in the liver from these mice by assessing the expression of iron regulatory protein transferrin receptor 1 (TfR1), which is inversely proportional to the cellular iron status [33]. Thus, consistent with the literature, a decrease in TfR1 levels in the HID and Fe-Dex mice compared to their corresponding standard iron diet (SD) and PBS-injected mice control livers (Figure 1B) confirms iron accumulation in the HID and Fe-Dex mice liver. Both the dietary iron overload and Fe-Dex-injected mice showed a remarkable upregulation of GLUT3 isoform compared to their respective control mice livers (Figure 1C,D).

**Figure 2:**
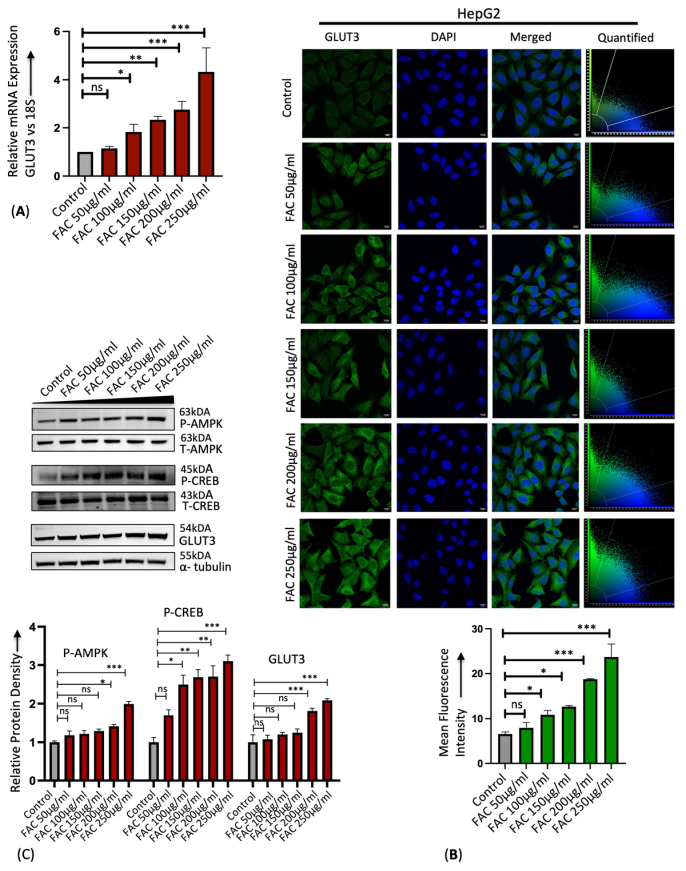
Iron upregulates hepatic GLUT3 expression in vitro in a dose-dependent manner. (**A**) Relative mRNA expression of GLUT3 by RT-PCR in HepG2 cells treated with different concentrations of FAC for 24 h. (**B**) Qualitative analysis of GLUT3 protein expression in FAC-treated HepG2 cells by confocal microscopy. Scale bar is 10 μm. Graph represents mean fluorescence intensity of GLUT3 expression. (**C**) Quantitative protein expression of p-AMPK (Thr-172), total AMPK, p-CREB (Ser 133), total CREB, and GLUT3 in HepG2 cells treated with FAC for 24 h by Western blot. α-tubulin was used as a loading control. Blots cropped from different parts of the same gel or from different gels are separated by white space. Data expressed as mean ± SEM of three independent experiments; *** *p* < 0.0001, ** *p* < 0.001, * *p* < 0.05.

Similarly, treatment of human liver cancer cell line HepG2 with iron in the form of ferric ammonium citrate (FAC) showed a dose-dependent increase in the GLUT3 mRNA and protein expression by RT-PCR and immunostaining (Figure 2A,B). Recently, GLUT3 has been reported to be significantly upregulated in colorectal cancer through AMP-activated protein Kinase (AMPK) and Cyclic AMP-Responsive Element-Binding protein 1 (CREB1) signaling [34]. It was shown that AMPK silencing by siRNA attenuated GLUT3 production and the activation of AMPK by 5-aminoimidazole-4-carboxamide riboside (AICAR)-upregulated GLUT3 transcription through CREB1 phosphorylation [34]. In addition, it was demonstrated through chromatin immunoprecipitation that CREB1 binds to the CREB1-binding site in the promoter region of GLUT3 and induces GLUT3 expression [34]. In this study, we found that iron treatment in HepG2 cells activated AMPK and CREB1 phosphorylation in a dose-dependent manner and induced GLUT3 expression in a Western blot (Figure 2C). Consistent with our findings in in vitro and in vivo studies, it is established that human hepatocellular carcinoma (HCC) exhibits elevated iron levels [8,35,36,37] and shows a significant upregulation of GLUT3 transcription compared to adjacent normal liver tissues [20,21]. These results indicate that iron overload significantly enhances hepatic GLUT3 expression.

### 3.2. Iron Enhances GLUT3 Expression through LKB1/AMPK/CREB1 Signaling

**Figure 3:**
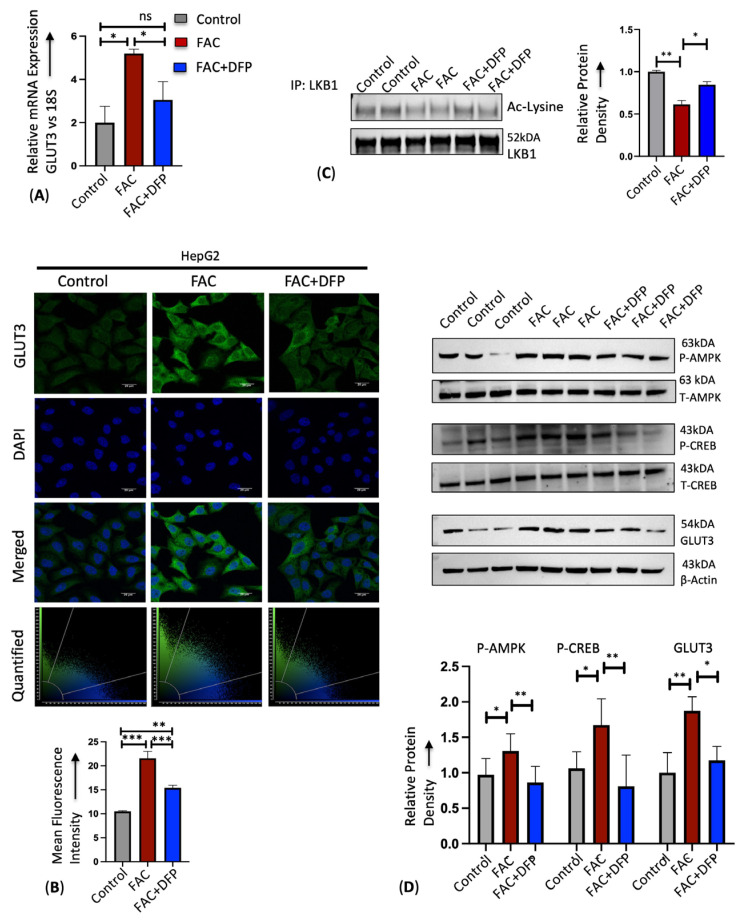
Iron enhances hepatic GLUT3 expression through LKB1/AMPK/CREB axis. HepG2 cells treated with PBS, 250 μg/mL FAC, or 250 μg/mL FAC co-treated with 100 μM DFP for 24 h were used. (**A**) Relative mRNA expression of GLUT3 was assessed by RT-PCR. (**B**) Qualitative analysis of GLUT3 protein expression by confocal microscopy. Scale bar is 20 μm. Graph represents relative intensity of GLUT3 expression. (**C**) Lysates from HepG2 cells were incubated and precipitated with LKB1 antibody. Pull down was analyzed by Western blot for acetylated lysine. LKB1 was blotted as a loading control. (**D**) Protein expression of p-AMPK (Thr-172), total AMPK, p-CREB (Ser 133), total CREB and GLUT3 were quantified by Western blot. Data expressed as mean ± SEM of three independent experiments; *** *p* < 0.001, ** *p* < 0.01, * *p* < 0.05, ns = non-significant.

We examined the mechanisms by which iron activated GLUT3 expression in the liver. We first confirmed that iron-mediated GLUT3 mRNA and protein upregulation is reversed in the presence of iron chelator deferiprone using RT-PCR and immunostaining (Figure 3A,B). The serine/threonine liver kinase B1 (LKB1) is a master kinase that phosphorylates and activates several kinases including AMPK [38]. A previous study demonstrated that a high-iron diet activates LKB1 in mice by increasing deacetylase and decreasing LKB1 acetylation [30]. Here, we found that HepG2 cells treated with iron enhanced LKB1 activity by decreasing LKB1 acetylation (Figure 3C) and consequently activated AMPK and CREB1 through phosphorylation, thereby upregulating downstream GLUT3 expression (Figure 3D). This iron-induced LKB1, AMPK, and CREB1 activation and GLUT3 upregulation was prevented in the presence of iron chelator deferiprone (Figure 3C,D).

### 3.3. GLUT3 Silencing Attenuates Iron-Mediated Cellular Hyperproliferation

**Figure 4:**
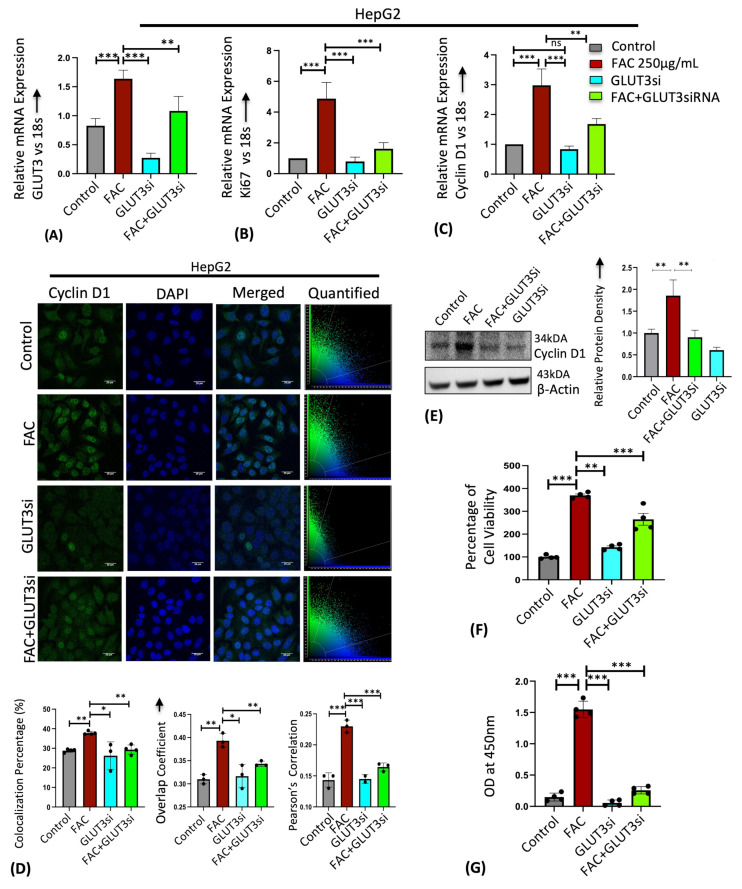
GLUT3 silencing prevents iron-mediated cellular hyperproliferation in HepG2 cells. FAC-treated HepG2 cells in the presence or absence of GLUT3siRNA were used. Relative mRNA expression of (**A**) GLUT3, (**B**) Ki67, and (**C**) cyclin D1 was assessed by RT-PCR. (**D**) Qualitative analysis of cyclin D1 protein expression by confocal microscopy. Graph represents relative intensity of cyclin D1 expression. Scale bar is 20 μm. (**E**) Quantitative protein expression of cyclin D1 by Western blot. Cell proliferation was quantified by (**F**) MTT assay and (**G**) BrdU assay. Data expressed as mean ± SEM of three independent experiments; *** *p* < 0.001, ** *p* < 0.01, * *p* < 0.05, ns = non-significant.

Malignant tumors have higher iron requirements for cell division and metabolic processes than non-malignant tumors [39]. Hence, iron overload is associated with several types of cancers, and iron chelators have been proven to reduce tumor cell proliferation [40,41]. Similarly, many studies have identified GLUT1 and GLUT3 upregulation to play a critical role in satisfying the energy needs of the tumor microenvironment [18,19,20,21]. To determine whether iron-induced cellular hyperproliferation is mediated through GLUT3 upregulation, we treated the hepatocellular carcinoma cell line HepG2 with iron in the presence or absence of small interfering RNA (siRNA)-mediated GLUT3 gene silencing and assessed the expression of cell cycle markers and cell proliferation. We first confirmed that GLUT3 expression was silenced by GLUT3-siRNA (GLUT3si) using RT-PCR (Figure 4A). Despite achieving a 50–60% reduction in GLUT3 expression with GLUT3 siRNA in HepG2 control cells, the effectiveness of siRNA was diminished in the presence of FAC. The efficiency of knockdown may vary due to multiple factors. Nevertheless, despite the reduced knockdown efficiency, siRNA treatment produced a significant phenotype in the FAC-treated HepG2 cells. Ki67, a nuclear antigen and a marker for active cell proliferation [42], was upregulated upon FAC treatment; however, this iron-mediated Ki67 mRNA upregulation was reversed when GLUT3 was inhibited by GLUT3si (Figure 4B). In addition, cyclin D1, a key protein involved in the regulation of cell proliferation and differentiation [43] and often augmented in human carcinomas, was upregulated with FAC treatment in RT-PCR, immunostaining, and Western blot, and this iron-induced cyclin D1 expression was prevented in FAC samples treated with GLUT3si (Figure 4C–E). In congruence with the expression of cell proliferation markers, GLUT3si significantly inhibited FAC-mediated cellular hyperproliferation, as shown in the MTT and BrdU cell proliferation assays (Figure 4F,G).

### 3.4. Sodium β-Hydroxybutyrate (BHB) Prevents Iron-Mediated GLUT3 Upregulation

**Figure 5:**
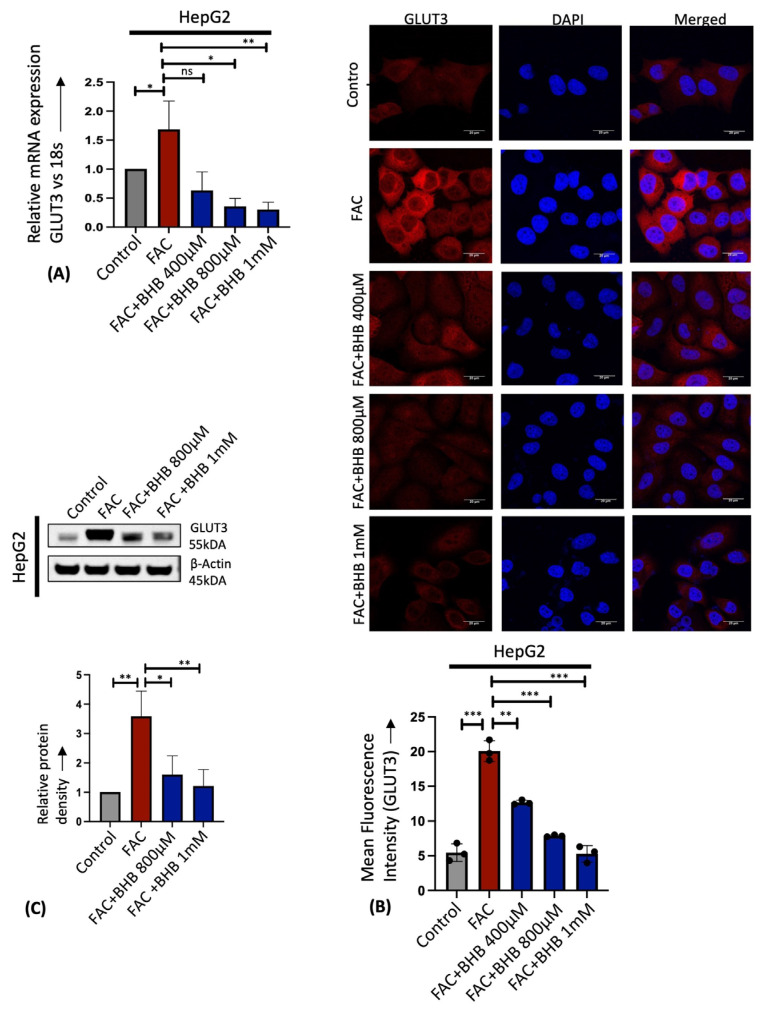
Treatment with BHB in vitro prevents FAC-induced GLUT3 upregulation in HepG2 cells. HepG2 cells treated with BHB at different concentrations for 24 h with or without FAC were used. (**A**) Relative mRNA expression of GLUT3 was assessed by RT-PCR. (**B**) Qualitative analysis of GLUT3 protein expression by confocal microscopy. Scale bar is 20 μm. (**C**) Western blot analysis of GLUT3 protein expression. Graph represents quantification of GLUT3 protein normalized to loading control by densitometry. Data expressed as mean ± SEM of three independent experiments; *** *p* < 0.001, ** *p* < 0.01, * *p* < 0.05, ns = non-significant.

**Figure 6:**
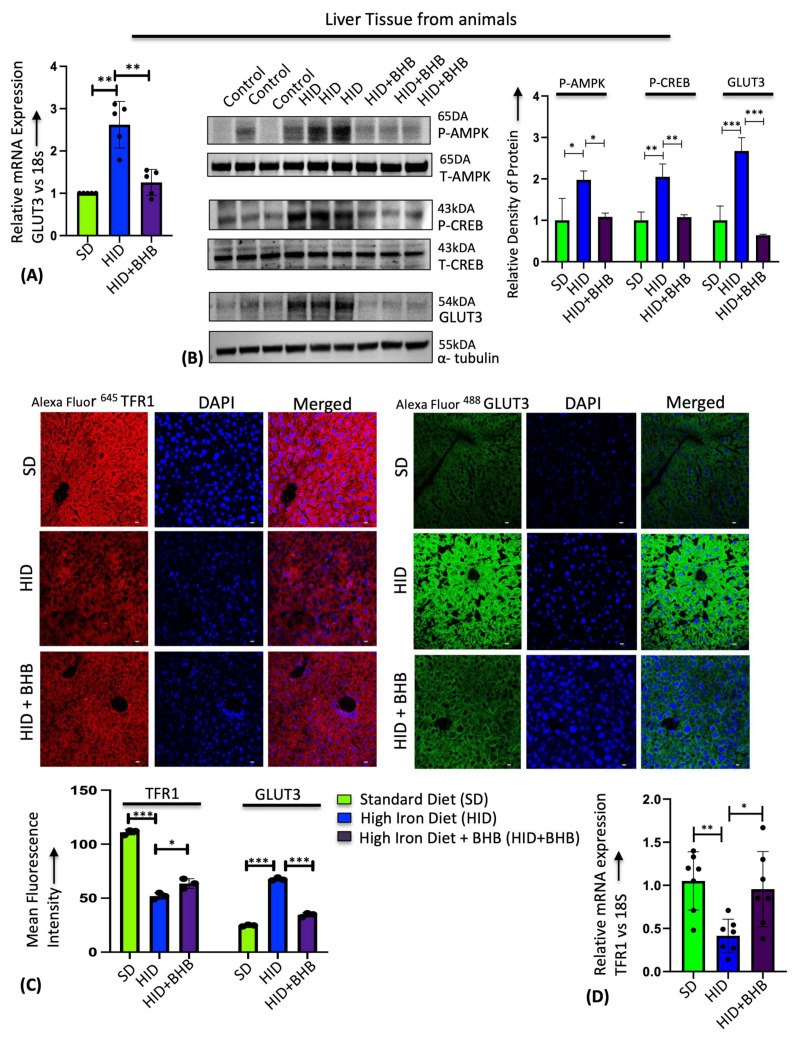
BHB treatment in vivo prevents iron-mediated hepatic GLUT3 upregulation in mice. HID-fed mice treated with or without BHB were used. SD-fed mice were used as control. (**A**) Relative mRNA expression of GLUT3 in the liver tissues from mice. (**B**) Protein expression of p-AMPK (Thr-172), total AMPK, p-CREB (Ser 133), total CREB, and GLUT3 by Western blot in the mouse liver tissues. Quantification of p-AMPK (Thr-172), p-CREB (Ser 133), and GLUT3 expression by densitometry. (**C**) Immunohistochemistry of TFR1 and GLUT3 expression patterns in the mouse liver tissues. Scale bar is 10 μm. Images were quantified to detect the mean fluorescence intensity of TFR1 and GLUT3 using Las X software version 3.5.19976.5. (**D**) Relative mRNA expression of TfR1 in the liver tissues from mice. Data expressed as mean ± SEM; n = 5 mice per group; *** *p* < 0.0001, ** *p* < 0.001, * *p* < 0.05, ns—not significant.

Ketone BHB inhibits the proliferation and viability of cancer cells but has no effect on healthy fibroblasts [44,45,46]. Here, we found that BHB treatment in HepG2 cells reversed the FAC-induced GLUT3 upregulation in a dose-dependent manner using RT-PCR, immunostaining, and Western blot (Figure 5A–C). Similarly, BHB treatment in mice on a high-iron diet prevented iron-mediated hepatic GLUT3 upregulation by RT-PCR (Figure 6A). This reduction in GLUT3 expression was accompanied by a reversal of the enhanced activity of AMPK and CREB1 by phosphorylation, as shown in the Western blot (Figure 6B). Further, immunostaining liver sections confirmed that iron levels were higher in the mice fed the high-iron diet as indicated by a reduction in TfR1 staining (Figure 6C left panel). Administration of BHB in conjunction with a high-iron diet partially restored TfR1 expression, although not fully to the levels observed in the control samples, as demonstrated by immunostaining and RT-PCR (Figure 6C,D). Consistent with the RT-PCR and Western blot, immunostaining confirmed that BHB treatment prevents iron-mediated GLUT3 upregulation in the liver (Figure 6C right panel), demonstrating the therapeutic potential of sodium BHB in preventing iron-mediated GLUT3 activation in vitro and in vivo.

## 4. Discussion

The prevalence and progression of cancer are associated with many risk factors like aging, genetics, diet, microenvironment, and metabolic factors [47]. Iron is one such element that has been attributed to exert carcinogenic activity by producing reactive oxygen species through the Fenton reaction, leading to mutagenesis and activation of proliferation pathways [35]. Thus, iron plays a critical role in all cancer stages including tumor incidence, growth, and metastasis [48,49,50]. Cancer cells reprogram themselves to increase intracellular iron uptake and retention by altering the expression of iron regulatory proteins in several types of cancer [51,52,53,54,55]. Also, systemic iron overload due to genetic, dietary, or lifestyle factors is known to significantly induce tumorigenesis [8]. In fact, an animal model fed an iron-rich diet resulted in the development of HCC directly in the absence of fibrosis or cirrhosis [36]. Similarly, hemochromatosis patients with HCC had higher rates of p53 mutations than other HCC patients [56], indicating the involvement of an important tumor suppressor gene, p53, in iron-mediated tumor progression. In addition, iron can act synergistically with other determinants like viral hepatitis, non-alcoholic fatty liver disease, and insulin resistance, contributing further to the pathogenesis of HCC [37]. In agreement with this phenomenon, removing iron from tumor cells, both in vitro and in vivo, by the treatment of iron chelators, dietary iron depletion, phlebotomy, and interference with the hepcidin–ferroportin axis has shown significant success in cancer therapy [57,58,59]. On the contrary, there are reports demonstrating decreases in tumor progression with increases in systemic or intracellular iron levels [60,61,62]. This paradox may indicate that cancer cells increase iron content to meet their metabolic demands during rapid proliferation but not to the levels that induce ferroptosis, a nonapoptotic form of cell death caused by iron-catalyzed lipid peroxidation of cell membranes, or that impair oncogenic signaling.

Cancer cells increasingly rely on glucose for their survival, with the glycolysis rate of tumor cells being over 200 times higher than normal cells [63]. Hence, cancer cells are more susceptible to apoptosis when glucose is deprived [16]. Incidentally, glucose transporters GLUT1 and GLUT3 are upregulated in several cancers like HCC, with their overexpression correlating with poor survival [18,19,20,21]. GLUT1 is a basal glucose transporter expressed in most of the cell types in the human body. The level of GLUT1 expression has been found to correlate with the degree of tumor incidence, migration, and invasion [16,64]. Further, treatment with anti-GLUT1 antibodies induced apoptosis in cancer cells, and this effect was additive when used with other clinically approved chemotherapeutic drugs [16,64]. However, GLUT1 may not be an ideal target for cancer therapy due to the global expression of GLUT1 in all cell types [17]. Hence, GLUT3, which is also upregulated in many cancers [13], would make a better therapeutic target, with its expression profile limited to specific cell types. Despite the intricate association of iron as well as glucose transporters GLUT1 and GLUT3 with cancer, there is limited knowledge on the role of iron in the expression of these glucose transporters. In the present study, we have shown that iron significantly upregulates GLUT3 expression in the liver. GLUT3 is primarily involved in the neuronal transport of glucose but is also found to be expressed at low levels in other organs, including the liver [13]. GLUT3 has the highest affinity for glucose compared to other class I glucose transporters [13]. In fact, inhibition of GLUT1 expression with GLUT1siRNA decreased the glucose uptake to a lesser extent than when GLUT3 was inhibited [65]. Further, overexpression of GLUT3 promoted tumor progression and metastasis [66,67,68], further highlighting its potential significance in therapeutic intervention. Surprisingly, like iron overload, iron depletion also has been demonstrated to induce GLUT1 expression and enhance glucose uptake in HepG2 cells [69]. Iron deficiency is associated with oxidative stress arising from mitochondrial dysfunction, whereas iron overload leads to oxidative stress through Fenton’s reaction. The common effects of both iron deficiency and iron overload on glucose transport via distinct molecular pathways reflect the complex interplay between iron status, oxidative stress, and metabolic regulation.

**Figure 7:**
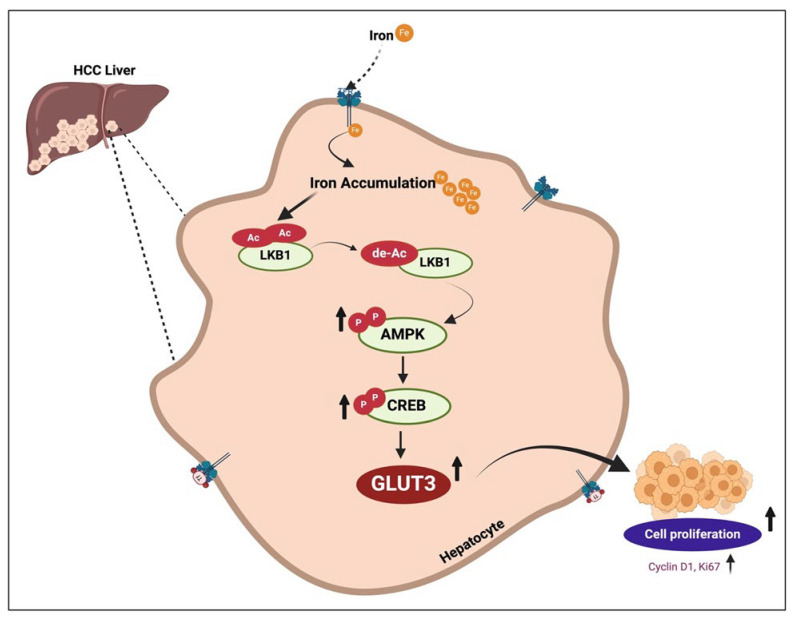
A schematic of the signaling pathways involved in iron-induced cellular hyperproliferation through GLUT3 upregulation in the liver.

Emerging evidence demonstrates a bidirectional relationship between iron and glucose metabolism [70]. In congruence with our results on GLUT3 upregulation by iron, mice fed a high-iron diet have been found to exhibit enhanced glucose uptake and AMPK phosphorylation [30], suggesting glucose as a preferential energy source during iron overload. Iron was shown to activate AMPK, a sensor of the cellular energy level, in turn suppressing gluconeogenesis by downregulating glucose-6-phosphatase, an enzyme that catalyzes the last reaction in gluconeogenesis [71]. A high-iron diet in mice was found to activate LKB1 by increasing deacetylase and decreasing LKB1 acetylation, which in turn upregulated AMPK phosphorylation [30]. Interestingly, in colorectal cancer, AMPK-siRNA attenuated GLUT3 production by downregulating CREB1 phosphorylation [34]. Further, CREB1 bound to the promoter region of GLUT3 and induced GLUT3 expression [34]. Here, we demonstrate that iron increases hepatic cell proliferation by enhancing GLUT3 expression through LKB1/AMPK/CREB1 signaling, as shown in Figure 7.

We found that exogenous treatment with ketone BHB prevented iron-mediated GLUT3 activation. A previous report established that unlike normal healthy cells, glioma cells failed to utilize BHB as an energy source during glucose deprivation [72]. Since ketones are metabolized in the mitochondria, cancer cells with impaired mitochondria may be unable to metabolize ketones effectively for energy [72]. In addition, sodium BHB has been demonstrated to impair cancer cell survival and proliferation by inhibiting glycolysis and energy production [73]. Importantly, BHB acts as an endogenous HDAC inhibitor eliciting anticancer effects by regulating the expression of oncogenes and tumor suppressor genes [74,75]. Interestingly, the expression of enzyme mitochondrial 3-hydroxybutyrate dehydrogenase 1 (BDH1), which catalyzes the final step in the production of endogenous BHB, has been reported to be significantly lower in HCC tissues compared to adjacent normal tissues [76]. In addition, ectopic BDH1 expression inhibited tumor proliferation and attenuated the migration and invasion of HCC cells in vitro [76,77].

BHB potentially attenuates GLUT3 upregulation induced by iron overload, partly through the reduction in hepatic iron levels. Consistent with our findings, previous studies have indicated that BHB reduces iron levels in the kidney and brain tissues [78,79]. However, the specific mechanisms underlying the BHB-mediated reduction in tissue iron levels require further investigation in future research. Our results open a new avenue for BHB as a potential therapeutic drug for several iron overload-associated chronic diseases. Future studies will aim to determine the effect of BHB on the expression of other isoforms of glucose transporters. The potential regulators of GLUT3 transcription and translation also remain largely unknown, warranting further research. Genetic approaches to generate conditional GLUT3-knockout mice and the identification of GLUT3-specific inhibitory compounds will be useful for the functional characterization of GLUT3 and the development of novel drugs for cancer therapy.

## Data Availability

The datasets generated and analyzed during the current study are available from the corresponding author on reasonable request.

## Supplementary Materials

The following supporting information can be downloaded at: https://www.mdpi.com/article/10.3390/cells13131147/s1, Figure S1: Cell proliferation quantified by MTT assay in HepG2 cells at different doses of GLUT3siRNA; Table S1: List of primers used for RT-PCR.
