# Supplementary material for: Iron Regulates Cellular Proliferation by Enhancing the Expression of Glucose Transporter GLUT3 in the Liver
Source: Cells. 2024 Jul 4;13(13):1147. doi: 10.3390/cells13131147 (PMC11240476; doi:10.3390/cells13131147)

Table S1: List of primers used for RT-PCR

Human

Primers	Forward Sequence	Reverse Sequence
H_GLUT1	5'-TTGCAGGCTTCTCCAACTGGACCTC-3'	5'-AACAGAACCAGGAGCACAGTGAAGA-3'
H_GLUT2	5'-TCATGTCAGTGGGACTTGTGCTGCTG-3'	5'-AAACTCAGCCACCATGAACCAGG-3'
H_GLUT3	5'-TGCCTTTGGCACTCTCAACAAGC-3'	5'-GCCATAGCTCTTCAGACCCAAGGAT-3'
H_GLUT4	5'-TGCCTTTGGCACTCTCAACAAGC-3'	5'-GCCATAGCTCTTCAGACCCAAGGAT-3'
H_TfR1	5'-GAGGACGCGCTAGTGTTCTT-3'	5'-TGTGACATTGGCCCTTGTGTT-3'
H_Cyclin D1	5'-TCTACACCGACAACTCCATCCG-3'	5'-TCTGGCATTTTGGAGAGGAAGTG-3'
H_Ki67	5'-GAAAGAGTGGCAACCTGCCTTC-3'	5'-GCACCAAGTTTTACTACATCTGCC-3'
H_18S	5'-GATTCCGTGGGTGGTGGTGC-3'	5'-AAGAAGTTGGGGACGCCGA-3'

Mouse

Primers	Forward Sequence	Reverse Sequence
M_GLUT1	5'-GCTTCTCCAACTGGACCTCAAAC-3'	5'-ACGAGGAGCACCGTGAAGATGA-3'
M_GLUT2	5'-CTGGGTCTGCAATTTTGTCA-3'	5'-TGTAAACAGGGTGAAGACCA-3'
M_GLUT3	5'-CTCTTCAGGTCACCCAACTACGTC-3'	5'-CGGGCAATGTCCTCGAAAG-3'
M_GLUT4	5'-GTCCTCCTGCTTGGCTTCTT-3'	5'-AGCTGAGATCTGGTCAAACG-3'
M_TfR1	5'-GGCAAGTAGATGGAGATAACAGTC-3'	5'-CTGAATGCCACATAACCCTC-3'
M_18S	5'-GCAATTATTCCCCATGAACG-3'	5'-GGCCTCACTAAACCATCCAA-3'

Supplementary Figure S1. Cell proliferation quantified by MTT assay in HepG2 cells at different doses of GLUT3siRNA.

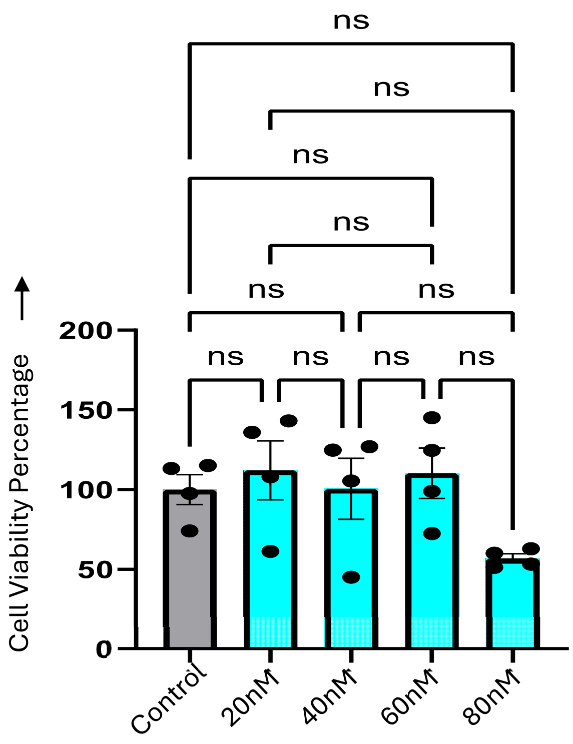

Supplement: Supplementary file 1 [file cells-13-01147-s001.zip › cells-3015859-supplementary.pdf]
